# Ten-Year Follow-Up of Mammography and Ultrasonography for Detection of Locoregional Breast Cancer Recurrence in Asian Female Patients

**DOI:** 10.3390/cancers18010064

**Published:** 2025-12-24

**Authors:** Joon Suk Moon, In Hee Lee, Byeongju Kang, Ho Yong Park, Hye Jung Kim, Won Hwa Kim, Yee Soo Chae, Soo Jung Lee, Jeeyeon Lee

**Affiliations:** 1 Department of Surgery, School of Medicine, Kyungpook National University, Daegu 41566, Republic of Korea; 2 Kyungpook National University Chilgok Hospital, Daegu 41404, Republic of Korea; 3 Department of Oncology/Hematology, School of Medicine, Kyungpook National University, Daegu 41566, Republic of Korea; 4 Department of Radiology, School of Medicine, Kyungpook National University, Daegu 41566, Republic of Korea

**Keywords:** breast cancer, mammography, ultrasound, recurrence, surveillance

## Abstract

Background: Breast cancer requires long-term surveillance, as recurrence and mortality risks extend beyond 10 years. Mammography (MMG) is the standard imaging modality; however, its effectiveness is reduced in Asian women owing to dense breast tissue. The optimal timing of surveillance MMG after breast-conserving surgery remains unclear, particularly the value of routine 6-month MMG. We evaluated the roles of MMG and ultrasound in detecting ipsilateral and contralateral breast cancer recurrence after breast-conserving surgery. Methods: This retrospective study included 961 patients with operable breast cancer who underwent breast-conserving surgery with or without axillary surgery between 2011 and 2015 at Kyungpook National University Chilgok Hospital. Surveillance consisted of biannual imaging for the first 2 years, followed by annual imaging for up to 10 years. Ipsilateral and contralateral breast cancer recurrences were analyzed according to detection modality, including MMG and ultrasound. Multivariate Cox proportional hazards regression analysis was performed to identify independent risk factors for recurrence. Results: During a mean follow-up of 139 months, 56 patients (5.8%) experienced locoregional recurrence, and 41 (4.3%) developed distant metastasis. Among 35 in-breast recurrences, 14 (40.0%) were ipsilateral and 21 (60.0%) contralateral. Ipsilateral recurrences were more often detected via either MMG or ultrasound alone, whereas contralateral cancers were typically detected through both modalities. During the first postoperative year, all ipsilateral and contralateral recurrences were detected exclusively by ultrasound, with no cancers identified by 6-month MMG (95% CI for 6-month detection: 0–0.38%). Multivariate analysis identified positive axillary lymph node status as the only independent predictor of locoregional recurrence (HR 2.52, 95% CI 1.14–5.54, *p* = 0.022). Detection patterns showed no significant differences across molecular subtypes (*p* = 0.665). Conclusions: Annual MMG remains appropriate for breast cancer surveillance in accordance with current guidelines. However, MMG at 6 months post-surgery may be unnecessary, as early detection during the first year was achieved solely by ultrasound. The complementary role of MMG and ultrasound is consistent regardless of molecular subtype.

## Simple Summary

This study examined the effectiveness of mammography and ultrasound in detecting locoregional recurrence among Asian women with breast cancer who underwent breast-conserving surgery. During 10 years of follow-up, both modalities showed comparable performance in identifying recurrences, although early detection within the first postoperative year was achieved exclusively by ultrasound. These findings suggest that annual mammography remains appropriate for surveillance, while early ultrasound is sufficient during the first year after surgery.

## 1. Introduction

Breast cancer is a heterogeneous disease, with prognosis, treatment strategies, and recurrence or mortality patterns differing by molecular subtype [1,2,3]. Approximately 60–70% of breast cancers are hormone receptor-positive. In patients who discontinued endocrine therapy after 5 years, long-term follow-up over 20 years demonstrated a persistent increase in recurrence and mortality risk, irrespective of nodal status [4]. Therefore, unlike many other malignancies, breast cancer necessitates extended surveillance of at least 10 years [5,6,7].

According to the National Comprehensive Cancer Network (NCCN) guidelines, post-treatment follow-up for patients with stage I–III breast cancer includes history and physical examinations 1–4 times per year for 5 years, and annually thereafter. Annual mammography (MMG) is also recommended, beginning 6–12 months after completion of surgery in patients who underwent breast-conserving surgery followed by radiation [8]. Similarly, the 11th Korean Clinical Practice Guideline for Breast Cancer recommends that patients who underwent breast-conserving surgery followed by radiotherapy should undergo MMG of the operated breast approximately 6 months after surgery, and then every 6–12 months for 2–5 years [9]. The contralateral, unaffected breast should be examined with annual MMG.

Dense breast tissue significantly reduces mammographic sensitivity and is an independent risk factor for breast cancer occurrence [10,11,12]. Maskarinec et al. reported that the mean unadjusted dense area was approximately 15% smaller in Japanese and Chinese women, whereas the proportion of dense tissue relative to total breast area was approximately 20% higher than that of Caucasian and Native Hawaiian women [13]. Younger women have denser breast [14,15]. Similarly, in screening MMG, over 50% of Korean women aged 40 years have dense breast [16]. However, most guidelines recommend uniform screening protocols irrespective of race.

MMG remains the standard screening modality for breast cancer; however, discomfort and pain during the procedure are common and may cause some women to discontinue screening [17,18,19]. Pain is often stronger in breasts that have undergone surgery or radiotherapy than in unaffected breasts [20,21]. Therefore, this study aims to investigate the distribution of imaging modalities used to detect ipsilateral and contralateral breast cancer recurrences to clarify the respective roles of MMG and ultrasound in treated and untreated breasts.

## 2. Materials and Methods

Between 2011 and 2015, 1364 female patients with operable breast cancer treated at Kyungpook National University Chilgok Hospital were retrospectively reviewed. All patients had histologically confirmed breast cancer by needle or excisional biopsy and underwent breast surgery with or without axillary surgery. Preoperative evaluation of tumor size, location, number of breast lesions, and axillary lymph node metastasis was performed using MMG, ultrasonography, and breast magnetic resonance imaging (MRI). Patients who underwent breast-conserving surgery, with or without axillary surgery, were included in this study. Exclusion criteria included mastectomy without reconstruction (precluding mammographic follow-up), age ≥ 80 years, absence of regular follow-up, examinations performed exclusively at outside institutions, fewer than five follow-up visits within the past 5 years, and diagnosis of lobular carcinoma in situ. Overall, 961 patients met the inclusion criteria. All patients completed standard adjuvant therapy, and surveillance was performed biannually during the first 2 years and annually for the subsequent 8 years. Routine surveillance was conducted using blood tests, tumor marker assays, MMG, breast ultrasonography, chest and abdominal CT, and bone scans. Breast MRI was performed when clinically indicated.

The clinical variables assessed included age at diagnosis, body mass index (BMI), clinical and pathologic tumor size, axillary lymph node metastasis, overall stage, neoadjuvant and adjuvant treatments, histologic type, and molecular subtype. These variables were retrospectively obtained from patient medical records. Molecular subtypes were categorized as hormone receptor (HR)-positive breast cancer, defined as tumors exhibiting estrogen receptor (ER) or progesterone receptor (PR) expression of 1% or greater; human epidermal growth factor receptor 2 (HER2)-positive breast cancer (ER- and PR-negative with HER2 gene amplification or protein overexpression); or triple-negative breast cancer (TNBC), which lacks ER, PR, and HER2 expression. A Ki67 index was classified as high if >15% of tumor cells showed nuclear immunoreactivity. Histopathological assessment of the four biomarkers was performed according to the ASCO/CAP 2016 guidelines [22].

Oncological outcomes, including locoregional recurrence, distant metastasis, and mortality, were evaluated over a 10-year follow-up period, and cumulative risks were calculated. The timing and anatomical sites of recurrence or metastasis were documented, along with the imaging modalities used for lesion detection. Within the group of patients with locoregional recurrence, breast and axillary recurrences were analyzed separately. In-breast recurrences were further classified as ipsilateral or contralateral. All recurrences and metastatic events were included in the cumulative risks analysis over time.

Univariate and multivariate Cox proportional hazards regression analyses were performed to identify independent risk factors for locoregional recurrence. Variables included in the multivariate model were age (>50 vs. ≤50 years), BMI (>25 vs. ≤25 kg/m^2^), T stage (T2/3/4 vs. Tis/T1), N stage (positive vs. negative), molecular subtype (HR+/HER2−, HER2-positive, TNBC), and treatment factors (neoadjuvant and adjuvant chemotherapy). Hazard ratios (HR) and 95% confidence intervals (CI) were calculated. Statistical significance was set at *p* < 0.05. For the observation that 0 recurrences were detected by mammography at the 6-month interval, the 95% CI for a binomial proportion was calculated using the Clopper-Pearson method. Detection patterns across stages and molecular subtypes were compared using chi-square test.

## 3. Results

The mean age of the 961 patients was 51.3 years (standard deviation (SD) ± 10.1), and the mean BMI was 23.7 kg/m^2^ (SD ± 1.2). The mean clinical and pathologic tumor size were 1.7 (SD ± 0.9) and 1.5 (SD ± 0.8), respectively. Approximately 24% (n = 230) of patients had metastatic axillary lymph nodes, and over half (n = 549) of patients had stage 0-I breast cancer. Neoadjuvant chemotherapy was administered to 60 patients (6.2%), and adjuvant chemotherapy to 446 patients (46.3%). Most patients (n = 907, 94.4%) received radiotherapy.

The most common tumor type was invasive ductal carcinoma (n = 760, 79.1%), with 78 cases (8.1%) classified as TNBC. Over a mean follow-up period of 139.0 months (SD ± 16.8), 56 patients (5.8%) developed locoregional recurrence, and 41 (4.3%) developed distant metastasis. Fifty-four patients (5.6%) died from breast cancer (Table 1).

**Figure 1:**
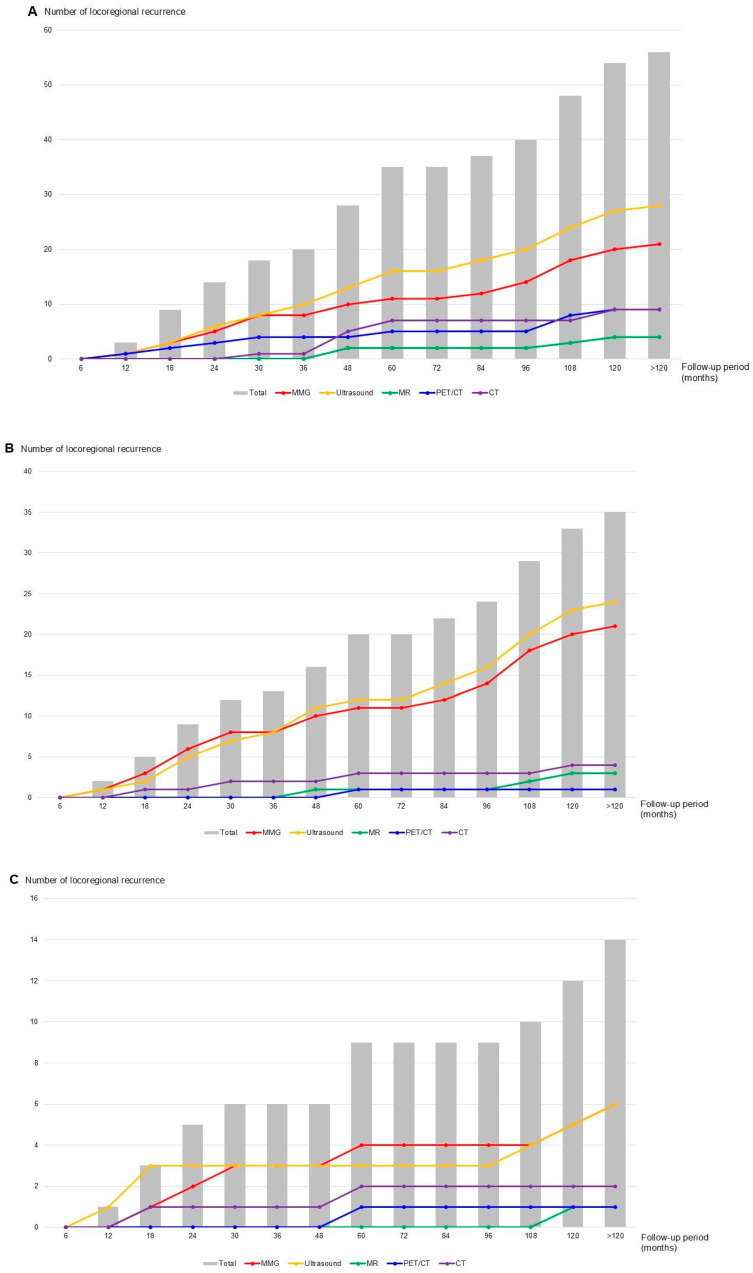
Distribution and detection patterns of local and locoregional recurrence by imaging modalities. (**A**) Rate of locoregional recurrence and types of imaging tests detected; (**B**) Rate of local recurrence and types of imaging tests found among patients with distant metastasis; (**C**) Proportion of local recurrences among patients who died and types of imaging tests found.

Among the 56 locoregional recurrences, 35 (62.5%) occurred in the breast, and 26 (46.4%) in the axillary region. Five patients experienced recurrence in both sites. During the 10-year follow-up, locoregional recurrence was most frequently detected by MMG and ultrasound. Detection rates were comparable during the first 30 months, after which ultrasound identified more recurrences (Figure 1A). Among patients with distant metastasis, locoregional recurrences were more often detected by MMG and ultrasound than by breast MRI. MMG identified slightly more recurrences within the first 36 months, whereas ultrasound detected more beyond 36 months. The 10-year cumulative risk showed a similar pattern for MMG and ultrasound (Figure 1B).

Among 35 cases of breast cancer recurrence, 21 (60.0%) were detected by MMG and 24 (68.6%) by ultrasound (Table 2). Of these, 14 cases (40.0%) were ipsilateral breast tumor recurrences, and 21 (60.0%) were contralateral breast occurrences (Table 3). No local recurrence was observed in the ipsilateral or contralateral breast during the first 6 months of follow-up. In ipsilateral recurrence, one case (8.3%) was detected exclusively by ultrasound within 12 months, one (8.3%) at 18 months exclusively by MMG, and one (8.3%) by chest CT. Of the five recurrences identified within 24 months, two (40.0%) were detected by MMG and three (60.0%) by ultrasound.

**Figure 2:**
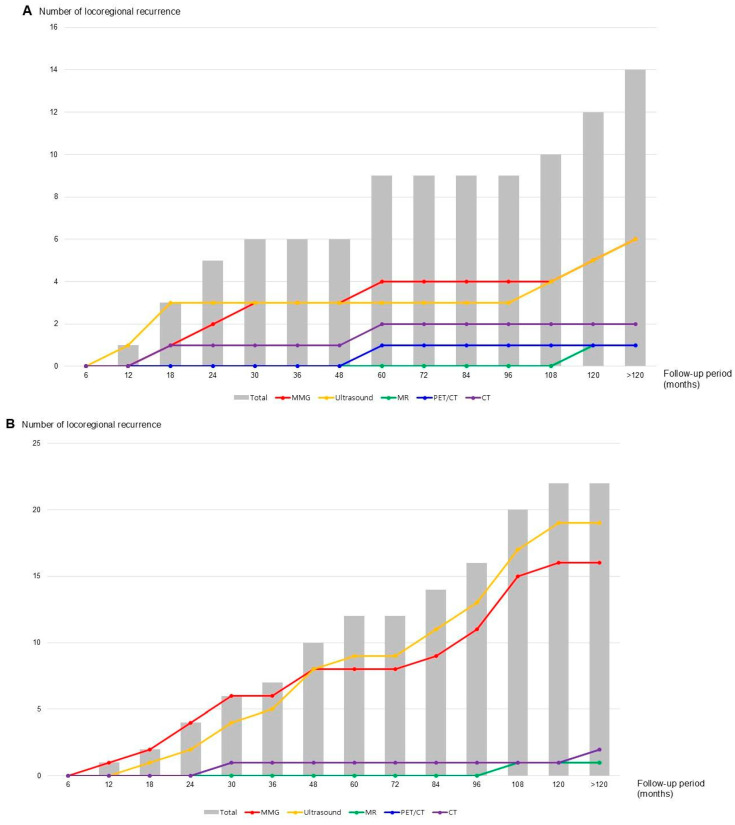
Cumulative cases of ipsilateral and contralateral breast recurrence according to imaging methods for detecting breast cancer recurrence. (**A**) Ipsilateral breast cancer recurrence; (**B**) Contralateral breast cancer occurrence.

In the 10-year cumulative analysis, ipsilateral breast cancer recurrence was more frequently detected by ultrasound than by MMG until 30 months, slightly more often by MMG after 48 months, and remained similar after 108 months. In contrast, contralateral recurrence steadily increased throughout the 10-year follow-up, with a high frequency of concurrent detection by MMG and ultrasound (Figure 2). During this period, four of the 14 ipsilateral cases (28.6%) were detected exclusively by MMG, while the remainder were identified through various imaging modalities. In contrast, only two contralateral recurrences (9.5%) were detected solely by MMG or ultrasound (Supplementary Figure S1).

The clinicopathologic characteristics and imaging detection patterns for the 56 patients with locoregional recurrence are summarized in Table 4. Consistent with the distribution of the overall cohort, most of recurrences were observed in patients with Stage IA disease (32/56, 57.1%) and the HR+/HER2− subtype (32/56, 57.1%). Among molecular subtypes, HR+/HER2− accounted for most recurrences (32/56, 57.1%), followed by TNBC (11/56, 19.6%), HR+/HER2+ (8/56, 14.3%), and HR−/HER2+ (5/56, 8.9%).

Among the 56 locoregional recurrences, imaging detection patterns showed complementary roles of MMG and ultrasound: 6 patients (10.7%) were detected by MMG only, 12 (21.4%) by ultrasound only, 14 (25.0%) by both MMG and ultrasound, and 24 (42.9%) through symptomatic presentation or other imaging modalities. When analyzing detection patterns by molecular subtype, HR+/HER2− patients showed relatively balanced detection rates between MMG (34.4%) and ultrasound (56.2%), with 25.0% detected by both modalities. TNBC patients demonstrated higher MMG detection (45.5%) compared to ultrasound (27.3%), with 27.3% detected by both. Statistical comparison revealed no significant differences in detection patterns across molecular subtypes (*p* = 0.665), indicating that the complementary role of MMG and ultrasound is consistent regardless of tumor biology.

Univariate and multivariate analyses were performed to identify independent risk factors for locoregional recurrence (Table 5). In univariate analysis, positive axillary lymph node status was significantly associated with locoregional recurrence (HR 2.45, 95% CI 1.22–4.92, *p* = 0.017).

In multivariate analysis adjusting for age, BMI, T stage, neoadjuvant and adjuvant chemotherapy, and molecular subtype, positive axillary lymph node status remained the only independent predictor of locoregional recurrence (HR 2.52, 95% CI 1.14–5.54, *p* = 0.022). Age, BMI, T stage, adjuvant chemotherapy, HER2-positive status, and TNBC were not significantly associated with recurrence risk in the multivariate model.

## 4. Discussion

Over a 10-year follow-up, ipsilateral breast cancer recurrences were more frequently detected by either MMG or ultrasound alone, whereas contralateral recurrences were typically identified by both modalities. The cumulative probability of ipsilateral recurrence detected by MMG was comparable between biannual and annual examinations. Our multivariate analysis identified positive axillary lymph node status as the only independent predictor of locoregional recurrence, with a 2.5-fold increased risk compared to node-negative patients.

The NCCN guidelines recommend annual MMG, noting that shorter screening intervals do not confer additional benefit. However, for patients who have undergone breast-conserving surgery with radiation therapy, the first post-treatment mammogram is advised 6–12 months after surgery [8]. A similar surveillance protocol is outlined in the clinical guidelines of the Korean Breast Cancer Society [9]. However, in real-world settings, many patients continue to experience breast swelling or pain from radiation therapy at 6 months, and surgical scars are probably not fully healed. In contrast, ultrasonography is less painful, can be performed immediately after surgery to evaluate postoperative complications, and generally causes minimal discomfort to patients.

Our finding that 0 recurrences were detected by mammography at the 6-month interval (95% CI: 0–0.38%) provides statistical confidence that early postoperative mammography may have limited clinical utility. In practical terms, even under a protocol that strongly recommended a 6-month MMG and achieved an adherence rate of 98.2%, MMG did not identify a single additional recurrence beyond those detected by ultrasound or subsequent imaging, indicating that its incremental diagnostic yield at this time point is extremely low. With a 98.2% adherence rate to the 6-month surveillance protocol, this lack of detection was not attributable to missed examinations. Wang et al. reported that among patients who underwent breast-conserving surgery after neoadjuvant chemotherapy, only 22 (15.8%) developed recurrence during 65 months of follow-up with MMG at 6-month intervals, of which only 8 (5.8%) were local recurrence [23]. In patients who underwent breast-conserving surgery followed by radiation therapy, a uniform surveillance strategy may be inappropriate. The risk of locoregional recurrence varies considerably by molecular subtype, with luminal A and B cancers showing very low recurrence rates, whereas TNBC and HER2-positive cancers demonstrate a considerably higher risk [24,25,26,27,28]. However, our stratified analysis by molecular subtype (Table 4) demonstrated that detection patterns were remarkably consistent across subtypes, with no statistically significant differences (*p* = 0.665). Among HR+/HER2− patients, mammography detected 34.4% and ultrasound detected 56.2% of recurrences. In TNBC patients, mammography detected 45.5% and ultrasound detected 27.3% of recurrences. These findings suggest that the complementary role of mammography and ultrasound is maintained regardless of tumor biology, supporting a uniform surveillance approach with respect to imaging modality selection. Within this framework, the absence of MMG-detected recurrences at 6 months and the exclusive contribution of ultrasound to early (≤12-month) detection indicate that increasing the frequency of MMG in the first postoperative year offers little additional benefit over ultrasound-based surveillance, even in biologically higher-risk subgroups

The identification of positive axillary lymph node status as the only independent predictor of locoregional recurrence has important clinical implications. Patients with node-positive disease may benefit from more intensive surveillance strategies, although the optimal intensification approach requires further investigation. Interestingly, molecular subtype was not an independent predictor in our multivariate model, suggesting that nodal status may be a more important determinant of recurrence risk than intrinsic tumor biology in the context of modern adjuvant therapy.

Asian women more frequently have dense breasts than Western women, which has been shown to reduce the effectiveness of mammographic screening [29,30,31]. In patients with breast cancer, ipsilateral recurrences should be monitored according to surveillance guidelines, whereas contralateral breast cancers can be managed with standard screening protocols. In this study, most contralateral breast cancers were detected by both MMG and ultrasound, while ipsilateral recurrences were more often identified by either modality alone. However, within the first year after surgery, both contralateral and ipsilateral breast cancers were detected exclusively by ultrasound. These findings suggest that, although annual MMG for breast cancer surveillance remains appropriate as recommended by current guidelines, the initial MMG may reasonably begin at 12 months after surgery rather than at 6 months.

This study has some limitations. First, its retrospective design precluded full standardization of surveillance protocols across all patients. In some instances, breast MRI was performed instead of ultrasound, and MMG was omitted in patients who experienced significant discomfort after surgery or radiation therapy. Second, although the study included over 10 years of follow-up, changes in imaging techniques and equipment performance over time, alongside the single-institution setting, may limit the generalizability of the findings.

Despite these limitations, this study represents one of the longest systematic follow-up analyses in breast cancer surveillance, with over 10 years of observation. The large sample size and comprehensive assessment of both ipsilateral recurrence and contralateral breast cancer detection across multiple imaging modalities enhance its clinical relevance, providing practical evidence to guide surveillance strategies in real-world settings.

## 5. Conclusions

In summary, this study supports annual MMG as an appropriate surveillance strategy for breast cancer, consistent with current guidelines. However, MMG performed at 6 months post-surgery may be unnecessary, as early postoperative detection within the first year was achieved exclusively by ultrasound. Given that the routine 6-month MMG examination did not detect any recurrences in 961 patients and that ultrasound captured all early events, our data support initiating mammographic surveillance at 12 months after surgery, in combination with ultrasound, rather than mandating an additional 6-month MMG in similar practice settings. Adapting surveillance strategies to individual patient characteristics, particularly nodal status, rather than applying a uniform approach, may enhance both effectiveness and patient comfort.

## Figures and Tables

**Table 1:** Demographics of patients with breast cancer who received breast conserving surgery.

Variables	N = 961	Variables	N = 961
Age at diagnosis (mean ± SD, years)	51.3 ± 10.1	Types of cancer (n, %)	
Body mass index (mean ± SD, kg/m^2^)	23.7 ± 1.2	Ductal carcinoma in situ	122 (12.7)
Clinical tumor size (mean ± SD, cm)	1.7 ± 0.9	Invasive ductal carcinoma	760 (79.1)
Pathologic tumor size (mean ± SD, cm)	1.5 ± 0.8	Invasive lobular carcinoma	23 (2.4)
Metastatic axillary lymph nodes (n, %)		Mucinous carcinoma	23 (2.4)
Yes	230 (23.9)	Metaplastic carcinoma	6 (0.6)
No	731 (76.1)	Others *	27 (2.8)
Overall tumor stage (n, %)		Estrogen receptor * (n, %)	
0	122 (12.7)	Positive	734 (76.4)
IA	427 (44.4)	Negative	227 (23.6)
IB	9 (0.9)	Progesterone receptor * (n, %)	
IIA	221 (23.0)	Positive	666 (69.3)
IIB	98 (10.2)	Negative	295 (30.7)
IIIA	62 (6.5)	HER2 * (n, %)	
IIIC	22 (2.3)	Positive	123 (12.8)
Neoadjuvant chemotherapy (n, %)	60 (6.2)	Negative	838 (87.2)
Adjuvant chemotherapy (n, %)	445 (46.3)	Triple-negative breast cancer (n, %)	78 (8.1)
Adjuvant radiotherapy (n, %)	907 (94.4)	Locoregional recurrence (n, %)	56 (5.8)
Adjuvant hormonal therapy (n, %)	747 (77.7)	Distant metastasis (n, %)	41 (4.3)
Mean follow-up period (mean ± SD, months)	139.0 ± 16.8	Expired (n, %)	54 (5.6)

* Others include rare histologic subtypes such as adenoid cystic, tubular, cribriform, medullary, and mixed carcinomas. * Estrogen, Progesterone receptor positive: expression ≥ 1%; HER2 positive: IHC 3+ or ISH-amplified.

**Table 2:** Cumulative cases of locoregional recurrence according to imaging methods for detecting breast cancer recurrence.

Follow-Up Period (Months)		6	12	18	24	30	36	48	60	72	84	96	108	120	>120
Locoregional recurrence (n = 56)	MMG	0	1	3	5	8	8	10	11	11	12	14	18	20	21
	Ultrasound	0	1	3	6	8	10	13	16	16	18	20	24	27	28
	MR	0	0	0	0	0	0	2	2	2	2	2	3	4	4
	PET/CT	0	1	2	3	4	4	4	5	5	5	5	8	9	9
	CT	0	0	0	0	1	1	5	7	7	7	7	7	9	9
	Total	0	3	9	14	18	20	28	35	35	37	40	48	54	56
In breast recurrence (n = 35)	MMG	0	1	3	6	8	8	10	11	11	12	14	18	20	21
	Ultrasound	0	1	2	5	7	8	11	12	12	14	16	20	23	24
	MR	0	0	0	0	0	0	1	1	1	1	1	2	3	3
	PET/CT	0	0	0	0	0	0	0	1	1	1	1	1	1	1
	CT	0	0	1	1	2	2	2	3	3	3	3	3	4	4
	Total	0	2	5	9	12	13	16	20	20	22	24	29	33	35
Axillary region recurrence (n = 26)	MMG	0	0	1	2	3	3	4	4	4	4	4	4	4	4
	Ultrasound	0	0	2	2	2	3	4	6	6	6	7	7	7	7
	MR	0	0	0	0	0	0	1	1	1	1	1	1	1	1
	PET/CT	0	1	2	3	4	4	4	4	4	4	4	4	5	5
	CT	0	0	1	1	1	1	4	6	6	6	6	9	10	10
	Total	0	1	5	7	9	10	16	20	20	20	21	24	26	26

Each case can be detected in two or more image modalities.

**Table 3:** Cumulative cases of ipsilateral and contralateral breast recurrence according to imaging methods for detecting breast cancer recurrence.

Follow-Up Period (Months)		6	12	18	24	30	36	48	60	72	84	96	108	120	>120
Ipsilateral breast (n = 14)	MMG	0	0	1	2	3	3	3	4	4	4	4	4	5	6
	Ultrasound	0	1	1	3	3	3	3	3	3	3	3	4	5	6
	MR	0	0	0	0	0	0	0	0	0	0	0	0	1	1
	PET/CT	0	0	0	0	0	0	0	1	1	1	1	1	1	1
	CT	0	0	1	1	1	1	1	2	2	2	2	2	2	2
	Total	0	1	3	5	6	6	6	9	9	9	9	10	12	14
Contralateral breast (n = 21)	MMG	0	1	2	4	6	6	8	8	8	9	11	15	16	16
	Ultrasound	0	0	1	2	4	5	8	9	9	11	13	17	19	19
	MR	0	0	0	0	0	0	0	0	0	0	0	1	1	1
	PET/CT	0	0	0	0	1	1	1	1	1	1	1	1	1	2
	CT	0	0	0	0	1	1	1	1	1	1	1	1	1	2
	Total	0	1	2	4	6	7	10	12	12	14	16	20	22	22

Each case can be detected in two or more image modalities.

**Table 4:** Clinicopathologic features and imaging detection patterns of 56 patients with locoregional recurrence.

	Total (N = 56)	Detected by MMG Only	Detected by US Only	Detected by MMG & US	Symptomatic/Others	*p*-Value
Initial Stage (n, %)						0.028
Stage 0 (DCIS)	2	1 (50.0)	0 (0.0)	0 (0.0)	1 (50.0)	
Stage I	32	5 (15.6)	8 (25.0)	11 (34.4)	8 (25.0)	
Stage II	16	0 (0.0)	2 (12.5)	1 (6.2)	13 (81.3)	
Stage III	6	0 (0.0)	2 (33.3)	2 (33.3)	2 (33.3)	
Molecular Subtype (n, %)						0.660
HR+/HER2−	32	3 (9.4)	10 (31.2)	8 (25.0)	11 (34.4)	
HR+/HER2+	8	1 (12.5)	2 (25.0)	2 (25.0)	3 (37.5)	
HR−/HER2+	5	0 (0.0)	0 (0.0)	1 (20.0)	4 (80.0)	
Triple-negative (TNBC)	11	2 (18.2)	0 (0.0)	3 (27.3)	6 (54.5)	
Adjuvant Treatment (n, %)						-
Chemotherapy	32	2 (6.3)	7 (21.9)	6 (18.8)	17 (53.0)	
Endocrine Therapy	43	4 (9.3)	12 (27.9)	11 (25.6)	16 (37.2)	
Anti-HER2 Therapy	13	1 (7.7)	2 (15.4)	3 (23.1)	7 (53.8)	

All the data are expressed as numbers (percentages).

**Table 5:** Univariate and multivariate analysis of factors associated with locoregional recurrence.

Variables	Univariate Analysis		Multivariate Analysis	
	HR (95% CI)	*p*-Value	HR (95% CI)	*p*-Value
Age (>50 vs. ≤50)	0.87 (0.49–1.53)	0.669	0.79 (0.44–1.42)	0.426
BMI (>25 vs. ≤25)	1.52 (0.86–2.66)	0.170	1.45 (0.81–2.59)	0.21
T Stage (T2 vs. Tis/T1)	0.82 (0.32–2.09)	0.825	0.47 (0.17–1.31)	0.149
N Stage (N+ vs. N0)	2.45 (1.22–4.92)	0.017	2.52 (1.14–5.54)	0.022
Subtype				
HR+/HER2− (Ref)	1	-	1	-
HR−/HER2+	1.77 (0.89–3.53)	0.141	1.63 (0.79–3.35)	0.185
Triple-negative (TNBC)	1.95 (0.67–5.70)	0.275	1.87 (0.61–5.79)	0.275
Neoadjuvant Chemotherapy (Yes vs. No)	2.30 (0.99–5.33)	0.077	2.35 (0.94–5.88)	0.069
Adjuvant Chemotherapy (Yes vs. No)	1.51 (0.88–2.62)	0.164	1.51 (0.81–2.83)	0.195

## Data Availability

The datasets generated and analyzed during the current study are not publicly available. However, they are available from the corresponding author upon reasonable request.

## Supplementary Materials

The following supporting information can be downloaded at https://www.mdpi.com/article/10.3390/cancers18010064/s1. Figure S1: Imaging methods used to detect each case of ipsilateral and contralateral breast cancer recurrence. Four cases of ipsilateral breast cancer recurrence and two cases of contralateral breast cancer were detected exclusively by mammography.

## Abbreviations

The following abbreviations are used in this manuscript:

NCCN	National Comprehensive Cancer Network
ER	Estrogen receptor
PR	Progesterone receptor
TNBC	Triple-negative breast cancer
MMG	Mammography
