# Supplementary material for: Ten-Year Follow-Up of Mammography and Ultrasonography for Detection of Locoregional Breast Cancer Recurrence in Asian Female Patients
Source: Cancers (Basel). 2025 Dec 24;18(1):64. doi: 10.3390/cancers18010064 (PMC12784814; doi:10.3390/cancers18010064)
Supplement: Supplementary file 1 [file cancers-18-00064-s001.zip › cancers-3934951-supplementary.pdf]

Supplementary Figure S1. Imaging methods used to detect each case of ipsilateral and contralateral breast cancer recurrence. Four cases of ipsilateral breast cancer recurrence and two cases of contralateral breast cancer were detected exclusively by mammography (*).

Ipsilateral breast cancer recurrence								Contralateral breast cancer occurence					
Case number	FU period (mo)	MMG	US	MR	PET/CT	CT	Case number	FU period (mo)	MMG	US	MR	PET/CT	CT
1	~ 12						1	~ 12	*				
2	~ 18	*					2	~ 18					
3	~ 18						3	~ 24	*				
4	~ 24						4	~ 24					
5	~ 24						5	~ 30					
6	~ 30	*					6	~ 30					
7	~ 60						7	~ 36					
8	~ 60						8	~ 48					
9	~ 60	*					9	~ 48					
10	~ 108						10	~ 48					
11	~ 120						11	~ 60					
12	~ 120						12	~ 84					
13	>120						13	~ 84					
14	>120	*					14	~ 96					
							15	~ 96					
							16	~ 108					
							17	~ 108					
							18	~ 108					
							19	~ 108					
							20	~ 120					
							21	~ 120					
